# Developing and validation of COVID-19 media literacy scale among students during the COVID-19 pandemic

**DOI:** 10.1186/s40359-023-01353-6

**Published:** 2023-10-06

**Authors:** Hanieh Jormand, Majid Barati, Saeed Bashirian, Salman Khazaei, Ensiyeh Jenabi, Sepideh Zareian

**Affiliations:** 1 Vice-chancellor for research and technology, of Health Education and Promotion, Hamadan University of Medical Sciences, Hamadan, IR Iran; 2 Education and Promotion, Department of Public Health, School of Public Health, Social Determinants of Health Research Center, Hamadan University of Medical Sciences, Hamadan, IR Iran; 3 Department of Public Health, School of Public Health and Social Determinants of Health Research Center, Professor of Health Education and Promotion, Hamadan University of Medical Sciences, Hamadan, IR Iran; 4 Department of Epidemiology, School of Public and Social Determinants of Health Research Center, Hamadan University of Medical Sciences, Hamadan, IR Iran; 5 Hamadan University of Medical Sciences, Hamadan, Iran; 6 Vice-Chancellor for Research and Technology, Zareian. Sepideh (MSc), Hamadan University of Medical Sciences, Hamadan, Iran

**Keywords:** Factor analysis, Students, Psychometric, Digital Society, Cyberchondria, COVID-19, Coronavirus

## Abstract

**Objective:**

This cross-sectional validation work evaluated the psychometric features of the COVID-19 Media Literacy Scale (C-19MLs) in Students.

**Methods:**

The study was conducted on 530 students from a medical university in Hamadan, Iran, who were recruited through a stratified cluster random sampling process in June-July 2020. Intraclass Correlation Coefficient (ICC) and internal consistency were used to assess the reliability. Moreover, CFA (Confirmatory Factor Analyses) and EFA (Exploratory Factor Analyses) were carried out to examine construction validity. CVR (Content Validity Ratio) and CVI (Content Validity Index) were used to examine the content validity.

**Results:**

According to the factor analysis, it was indicated that the C-19MLs included 21 items measuring five dimensions (constructedness of credible Covid-19 media messages, contractedness of fake media coronavirus messages, fake media coronavirus messages, audience, with three questions in each factor; format, represented lifestyles in fake media coronavirus messages with six questions in each factor) for an explanation of 58.4% of the prevalent variance. The average scores for the CVI and CVR were respectively 0.94 and 0.77. According to confirmatory factor analysis, the studied model had an appropriate fitting to the data; the relative chi-square (x2/df) = 2.706 < 3, RMSEA = 0.093 ≤ 0.1; CFI = 0.893 ≥ 0.9; TLI = 0.874 ≥ 0.9; GFI = 0.816 ≥ 0.9; and SRMR = 0.06 ≤ 0.08. Further analyses represented acceptable findings for internal consistency reliability values with 0.86 of Cronbach’s alpha.

**Conclusions:**

The results proved that the C-19MLs is a reliable and valid tool, and it is suitable and acceptable now and can be utilized in forthcoming investigations. This highlights educators and stakeholders to realize the importance of participating individuals in the new media ecology and new ‘Infomedia’ ecosystems for enabling people in the current digital society.

**Supplementary Information:**

The online version contains supplementary material available at 10.1186/s40359-023-01353-6.

## Introduction

### The need to acquire media literacy skills

Media literacy is accessing, analyzing, evaluating, and conveying information in several printed and non-printed media [1]. Media literacy is the cognitive process utilized in critical thinking [2].

The rapid development of media technology in our daily lives causes the novel mode of creating and consuming information particularly attractive to adolescents as space and a platform for activities impossible in face-to-face communication [3, 4]. Media literacy skills can cope with their own media activities and expose themselves intentionally to the media [5]. Moreover, examining the results of meta-analysis investigations of the effectiveness of educational media literacy interventions on the avoidance of performances with high risks [6, 7] presents the facts of the need to implement health interventions to promote media literacy In Iran [8].

### A focus on media literacy related to coronavirus

Coronavirus (COVID-19) has been hotly discussed worldwide since January 30, 2020, as an international public health concern [9]. During this period, the youth are confined in their homes in particular situations, and many use social media apps for entertainment. These new opportunities facilitate access to a large body of information for learning and social interaction [10] while exposing them to dangerous individuals, including those with physical and mental health disorders. Based on several surveys, compulsive media use can be represented as an environmental risk factor for youth’s health, including having a sedentary lifestyle [11] or lower life satisfaction [12], anxiety, sleep disturbance [13], and stress [14].

Also, concerning all the draconian measures for the prevention of COVID-19, enabling individuals with media literacy and critically thinking about reports of media, truth or not true, and act using all forms of communication related to COVID-19 and have independent individual decision-making without interference from other environmental factors (media contents) to prevent the coronavirus disease [15, 16] is essential [17].

### Adopted theoretical framework of C-19MLs

In addition, there is a global need to study individuals’ health-specific media literacy competence [18], especially on specific pandemic issues [19]. On the other hand, one globally critical need, consultation, and recommendation for further action by the World Health Organization (WHO) and ”a key component of the COVID-19 global response” is increasing COVID-19 infodemic management in individuals [20]. To validate a measure of COVID-19 Media Literacy (C-19ML), applying an appropriate conceptual framework to evaluate the effectiveness of media literacy tools and the designed interventions is necessary. In this regard, the Media Literacy Training Center of the American CML (Theory CML Media Lit Kit) conceptual framework was selected [21], which is presented as a promising tool and evaluates the effectiveness of media literacy interventions [22–25]. Based on this framework, media literacy has five domains: “purpose, constructedness, audience, format, filter, and omit” [21]. Purpose means most media messages are organized to gain profit and power; “Why is this message being sent?“. The meaning of contractedness is that all media messages are constructedness;“ Who created this message?“. Audience means different people experience the same media message differently; “How might different people understand this message differently from me?“. Format and filter mean media messages are constructed using a language with its rules; “What creative techniques are used to attract my attention?“. The meaning of omitting is that media have embedded value and point of view; “What lifestyle, value and point of view are presented in or omitted from this message?”. These dimensions are the five core concepts of the selected framework [21]. Notably, this framework was used in other and near the scope of the present study [22, 24, 25].

Hence, the current study aimed to assess the psychometric features of C-19ML for measuring the youth’s C-19ML.

### Existing instruments and gaps

Several general and specific tools measure media literacy [16, 18, 19, 26, 27]. Each of these tools has introduced dimensions for media literacy (ML). These tools suffer from their narrow scopes and lack of tools targeted at measuring ML in specific issues to assess media literacy comprehensively in multidimensional skills. Moreover, the multidimensional specific media literacy to measure media literacy has mainly been neglected. For example, Chen et al. argue that media literacy is both an understanding producer and a consumer of media content [28]. Buckingham et al. emphasized functional and critical literacy, which define functional media literacy as competencies tools for critical thinking about creator media messages and understanding them at the textual level [29].

In contrast, critical media literacy is the ability to analyze and judge media messages and understand them at various contextual levels [28]. So, these notions about media literacy are traditional [30]. Recently, Li X et al. went beyond and developed a study to construct digital skills scales for primary and middle school children, especially in developing countries [4].

On the other hand, to the best of our knowledge, the number of studies proposing a tool to operationalize media literacy related to emerging diseases is limited [31]. Although some studies have been conducted using the researcher-made questionnaire about media literacy in Iran [16, 32], no prior research has psychometrically evaluated the C-19ML skills in individuals.

Also, several organizations such as the American Academy of Pediatrics, Center for Disease Control and Prevention, European Commission, UNESCO, and European Parliament and several media organizations such as Center for Media Literacy (CML), National Association for Media Literacy Education (NAMLE), and Association for Media Literacy (AML) have vigorously discussed the media literacy [20, 22, 23, 25]. Instead, several studies on Western countries’ media literacy or digital literacy scale [4].

Hence, the current work aimed to assess C-19ML’s psychometric features among college students in Iran, a developing country, and fill the empirical literature gap.

## Methods

### Statement

The Ethics Committee of Hamadan University of Medical Sciences approved this study with all consent processes (No: IR.UMSHA.REC.1399.229). Informed consent was obtained from all students; they were informed about the confidentiality of the information, the project’s purpose, and their voluntary participation in the study. All methods were performed based on relevant guidelines and regulations. The confidentiality of the information of the students was also assured. Informed consent was obtained from all the contributors.

### Instrument and item development

**Fig. 1:**
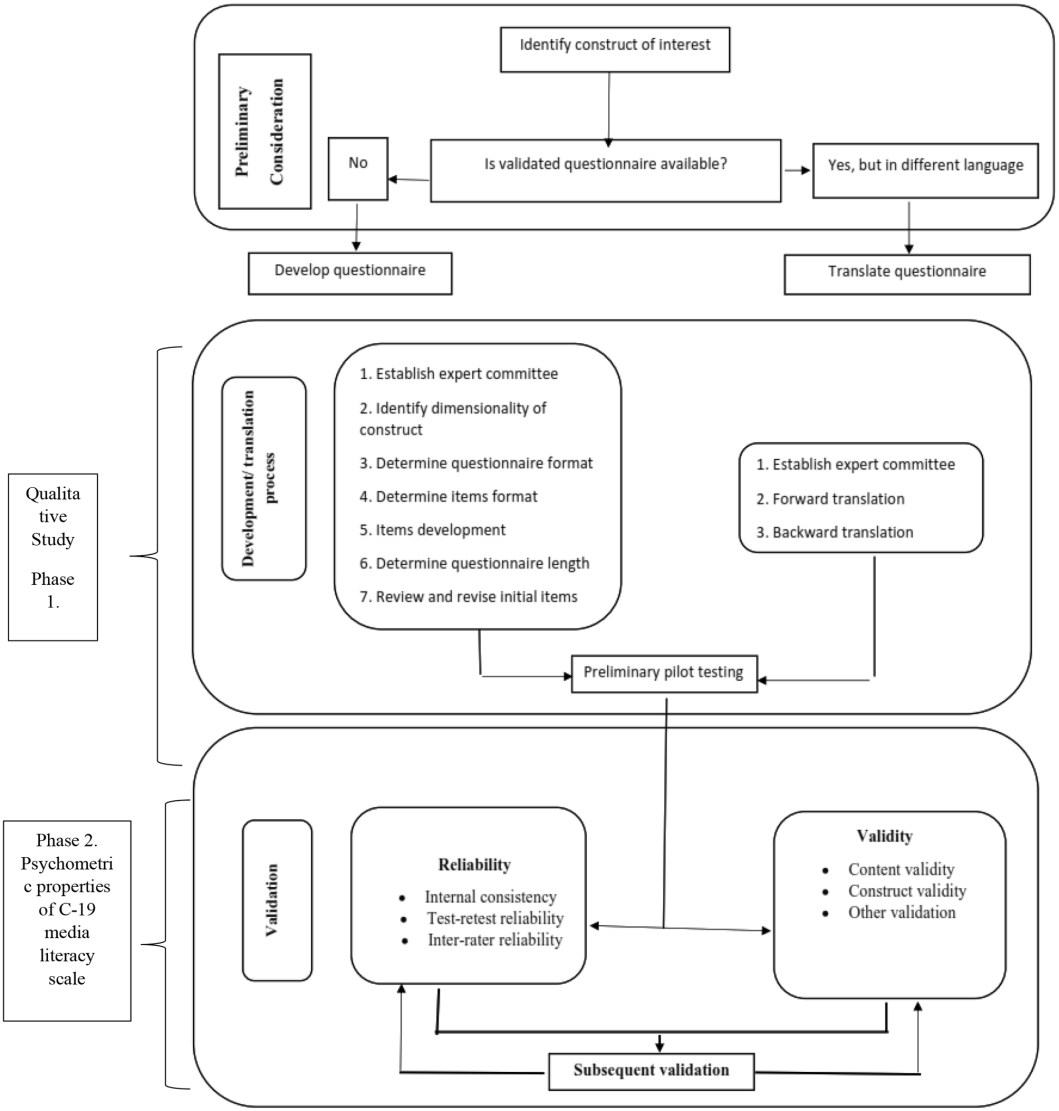
Processes of questioner development and validation [67]

In behavioral research, scale development begins with a thorough understanding of the concept to be measured [33]. So, this measurement instrument’s framework was defined in terms of the Media Literacy Training Center of the American CML[21]. Because no scale exists that measures the C-19 media literacy concepts in the student populations, the concept analysis process begins with exploring COVID-19 media literacy through interviews with people familiar with this concept and personal experience [15]. Then proceeds to write the items that were done [33]. The following processes of questionnaire development and validation [34] are offered in Fig. 1.

Based on the results of the previous qualitative study, the concept identification and concept analysis process was done concerning the appearance of new media technology along with emerging diseases, critical thinking about new concepts such as creators of media massages, methods of persuading the audience by creators of media messages and presented/ omit new patterns in lifestyle by misinformation and fake news were identified. So, generally, five concepts or factors consisting of (a) purpose, (b) contractedness, (c) audience, (d) format, and (e) filter and omit were identified [15]. Then, the items were completed by creating a matrix that visually displays the content to be addressed by the items and the dimensions of the concept that would be measured. This matrix helped the researcher identify the number and type of items required to measure the concept adequately. However, for the phrasing of items, the researcher turns to qualitative interviews with people familiar with this concept [33]. Results of the previous study showed that dimensions were required to adequately measure these concepts with the increased number of items of format and technique (8 items) and lifestyles represented in fake media coronavirus messages (8 items). Finally, the items of C-19MLs were extracted from 33 items measured on a 5-point Likert-type scale with “1 strongly disagree” and “5 strongly agree”.

### Psychometric properties of the scale

#### Face validity

The face validity of the C-19MLs was evaluated quantitatively and qualitatively.

#### Qualitative face validity evaluation

The face validity of the C-19MLs was evaluated qualitatively through the invitation of ten students to assess and comment on the items’ difficulty, suitability, vagueness, and relevancy. In this phase, the time needed for the implementation of this scale was adjusted. The scale amendments were made in terms of the student’s remarks.

#### Quantitative face validity evaluation

C-19ML’s quantitative face validity was evaluated to adopt the item impact method. Hence, ten students were requested to pilot the scale and determine the item’s rank over a Likert-based scale from 1 (non-important) to 5 (completely important). The effect of every item was ranked as the percentage of frequency multiplied by the importance. The frequency represents the number of students reporting a score of 4–5 to the considered item, and the importance was 4 or 5. When the effect score of an item was higher than 1.5, the item was regarded as appropriate and represented on a scale [35–37].

#### Content validity evaluation

The content validity of the C-19MLs was quantitatively and qualitatively evaluated.

#### Qualitative content validity evaluation

The content validity was calculated by delivering C-19MLs to 10 experts in health education (ten health education Ph.Ds.). They were then asked to present comments and assessments on the item allotments, phrasing, and ranking of the items [38]. The C-19MLs were revised in terms of feedback and comments.

#### Quantitative content validity evaluation

CVR (Content Validity Ratio) and CVI (Content Validity Index) were calculated quantitatively. CVR assesses the vitality of every item for the Iranian culture with a 3-point ranking scale (vital, valuable but not vital, and not vital**)** [39]. The CVR for every item was calculated by CVR = (Ne − (𝑁/2)) / (𝑁/2). Where Ne represents the number of panelists revealing “vital” for each definite item and N shows the overall number of panelists. Utilizing the Lawshe table, the CVR value is numerically obtained, in which a value of 0.62 is adopted for 10 panelists [40]. Using the ordinal scale with four possible responses, CVI was determined for simpleness, relevance, and clearness of every item. The answers included a score of 1 for not simple, unrelated, and unclear to 4 for very simple, very related, and very clear. The students’ judgment about the item as related or clear (with a rate of 3 or 4) was divided by the number of content specialists. CVI = 0.79 was suggested in some studies as a satisfactory lower limit [41, 42].

#### Target population and a sample

Thus, the target population in this work included students attending bachelor’s degree (BS) education to Ph.D. education. However, in the current research, 530 students from a medical university in Hamadan, Iran, were recruited for a stratified cluster random sampling process in June -July 2020 due to the coronavirus crisis in the Iran context.

Noticeably, the target population for conducting an exploratory factor analysis included 330 students from medical universities, who were recruited through a stratified cluster random sampling process, and the target population for performing confirmatory factor analysis included 200 students from a medical university who were recruited through a stratified cluster random sampling process and finally a total of 530 students from a medical university in Hamadan, Iran, were recruited for a stratified cluster random sampling process in June-July 2020.

#### Process and ethical considerations

The current research received approval (No: 9,904,102,236 and special ID of the Ethics Committee: IR.UMSHA.REC.1399.229) from the official review board and Ethics Panel at Hamadan University of Medical Sciences.

#### Procedures

Hamadan City is located in the west of Iran. It has one state medical university, Hamadan University of Medical Sciences. In the present study, strata were schools, and clusters were classes. In the first stage, each stratum has probability proportional to its size. So, eight schools were selected. In the second stage, in these eight schools, three classes were selected, and then 530 students were randomly selected with heterogeneous backgrounds and educational states.

Then, after coordinating with administrators of universities and colleges and obtaining their consent, they were referred to the students if they wanted to interview; aware that written consent was taken from them, they were assured that the information was confidential and then proceeded to collect the information. The inclusion criteria were to be a student of Hamadan University of Medical Sciences, interested in participating, and capable of responding and participating in the work and evaluation of Social Networks and the Internet. If any student wasn’t willing to not contribute to the study, he/she was excluded.

#### Measures

The students were asked to complete the C-19MLs questionnaires with two sections: [1] items representing the demographic data and [2] the C-19MLs (COVID-19 Media Literacy Scale).

The demographic questionnaires involved items of Sex, Age, Level of education, marital status, Major, Living status, Job status, Inspiration to use social media apps, and time spent utilizing the social media apps.

#### Measurement scales

Overall, 33 items were extracted for the variables mentioned above. The students were requested to evaluate their quantities of C-19MLs from 1 to 5, considering the items (1 for the minimum, 5 for the maximum) on a Likert-type scale. The sample items are “the objective of creating COVID-19 Media Messages to increase health literacy and self-caring in persons ”, “the WHO is among the Constructedness of credible messages about COVID-19”, “unproductive individuals are among the audience of coronavirus fake media messages,,” “Teaching simple preventive instructions and guidelines for public health Such as using frequent hands washed with ordinary soap and water, wearing a mask are utilized for attracting the attention of the audience incredible messages about Covid-19”, “In coronavirus, fake media messages, often represented in effects of drinking alcohol can prevent infection with the coronavirus.”

C-19MLs uses a five-item Likert-kind scale within the range of completely disagree [1] to completely agree [5]. The items and scores range for every subscale included: Purpose with four items, 4–20 scores range, Contractedness with six items and 6–30 scores range, Audience with seven items and 7 to 35 scores range, format with eight items and 8–40 scores range, and Represent Lifestyles with eight items and 8–40 scores range. The higher scores indicated a higher C-19ML.

#### Statistical analysis and Validity Assessment

The C-19MLs were validated based on content validity, construct validity, as well as face validity. Thus, the structural validity of the scale was examined using Exploratory Factor Analysis (EFA) with Promax rotation. A factor analysis was conducted by choosing a minimum sample size of 5–10 times the amount per item of the popular instrument [43]. Thus, the target population in this work included 330 students from medical universities in Hamadan, Iran, who were recruited through a stratified cluster random sampling process.

The Kaiser-Meyer-Olkin (KMO) and Bartlett’s Test of Sphericity were used to determine the appropriateness of the sample for factor analysis. Eigenvalues above one and factor loadings greater than 0.40 were considered appropriate to verify the possible underlying factors [43–46]. Furthermore, confirmatory factor analysis was performed with (AMOS Graphics, version 24. Thus, the target population in this work included 200 students from a medical university in Hamadan, Iran, who were recruited through a stratified cluster random sampling process based on some surveys that have recommended that this phase should be achieved on sample sizes between 100 and 200 participants [47]. Several goodness-of-fit indicators, including the chi-square ratio (χ2/df), the goodness of fit index (GFI), the root mean square error of approximation (RMSEA), standardized root mean square residual (SRMR), were selected for reporting the analysis outcomes. The following thresholds were considered to verify the model’s goodness of fit: χ2/df < 2.0, CFI, NFI, NNFI, and GFI ≥ 0.90–0.95, SRMR ≤ 0.05–0.08, and RMSEA ≤ 0.05–0.1 [48, 49].

Two types of validity are carried out for construct validity: convergent validity and discriminant validity. Average variance explained (AVE), construct reliability (CR), and maximum shared variance (MSV) are computed for all factors and are presented in Table 4. The AVE for each construct should be greater than 0.50; CR should be more than 0.7; CR is expected to be greater than AVE [50].

### Reliability evaluation

Ultimately, construct reliability, test-retest analyses, and internal consistency were utilized to assess C-19MLs’ reliability. For descriptive studies, Cronbach’s alpha coefficient was used to measure internal consistency. The tool’s reliability was measured by examining the internal consistency by Cronbach’s alpha coefficient. The stability was assessed by estimating the Intraclass Correlation Coefficient (ICC).

Alpha values ≥ 0.50 were regarded as satisfactory. The consistency levels were inferred by choosing the following class, in which α of 0.5 or less was considered improper, 0.50–0.60 as poor, 0.60–0.70 as moderate, 0.70–0.80 as good, 0.80–0.9 as very good, and higher than 0.90 was considered as excellent [36]. For ICC, thirty participants were randomly selected for completion of the scale after 2–4 weeks initially. A comparison was made for test-retest scores for every construct with the Pearson correlation test. The ICC values higher than 0.40 were regarded as acceptable. The consistency levels were inferred by choosing the following class: ICCs of 0.4 or lower were taken as poor to fair, 0.41–0.60 as moderate, 0.61–0.80 as acceptable, and higher than 0.80 as excellent [36].

## Results

### Sample features

In total, 530 students contributed to this work. The mean age of the respondents was 23.4 ± 5.22. Based on the educational status, Among the 530 participants, 369 participants (69.6%) mainly had bachelor’s degrees (BS), 43 participants (8.1%) had master’s degrees (MS), and 118 participants (22.3%) had Ph.D. Also, 352 participants (66.0%) were women, and 178 (83.6%) were single. Around 292 (55.1%) students lived in the dormitory, and 36 (6.8%) lived in student houses. Moreover, 160 (30.2%) of 530 participants with high social media app accessibility were high users (over 10 h), and 207 students (39.1%) used the internet moderately (for 3–6 h). (Table 1).

**Table 1:** Summary statistics for characteristics of study participants (n = 530)

Variables	EFA Stage (n = 330) Participants	CFA Stage (n = 200) Participants	Total Participants (n = 530)
Age (years), mean (SD)	23.95 (5.56)	23.42 (4.61)	23.74 (5.22)
Age, n(%)			
< 20	79 (23.9)	45 (22.5)	124 (23.4)
20–25	184 (55.8)	125 (62.5)	309 (58.3)
26–30	30 (9.1)	16 (8.0)	46 (8.7)
31–35	14 (4.2)	6 (3.0)	20 (3.8)
36–45	20 (6.1)	6 (3.0)	26 (4.9)
> 45	3 (0.9)	2 (1.0)	5 (0.9)
Gender, n(%)			
Men	106 (32.1)	72 (36.0)	178 (33.6)
Woman	224 (67.9)	128 (64.0)	352 (66.4)
Marital Status, n(%)			
Single	276 (83.6)	167 (83.5)	443 (83.6)
Married	54 (16.4)	33 (16.5)	87 (16.4)
Educational status, n(%)			
B.S Student	202 (61.2)	167(83.5)	369 (69.62)
MS Student	33 (10.0)	10 (5.0)	43 (8.11)
Ph.D. Student	95 (28.8)	23 (11.5)	118 (22.26)
School, n(%)			
Medical school	57 (17.3)	55 (27.5)	110 (20.75)
Health school	81 (24.5)	15 (7.5)	96 (18.11)
Pharmacy school	25 (7.6)	23 (11.5)	48 (9.06)
Paramedical school	50 (15.2)	34 (17.0)	84 (15.85)
Nursing school	74 (22.4)	39 (19.5)	113 (21.32)
Dentist school	20 (6.1)	20 (10.0)	40 (7.55)
Rehabilitation School	17 (5.2)	13 (6.5)	30 (5.66)
Other	6 (1.8)	1 (0.5)	7 (1.32)
Living states, n(%)			
Dormitory	181(54.8)	111(55.5)	292 (55.09)
lived in Hamadan city	101 (30.6)	57 (28.5)	158 (29.81)
lived in the Student Suite	21 (6.4)	15 (7.5)	36 (6.79)
other	27 (8.2)	17 (8.5)	44 (8.30)
Social Media Apps Accessibilities, n(%)			
low(1–2 h)	30 (9.1)	4 (2.0)	34 (6.42)
medium(3–6 h)	139 (42.1)	68 (34.0)	207 (39.06)
high(7–10 h)	88 (26.7)	72 (36.0)	160 (30.19)
very high(over 10 h)	73 (22.1)	56 (28.0)	129 (24.34)
Motivation on social media apps used ^a^, n(%)			
entertainment	178 (53.9)	122 (61.0)	300 (56.60)
Get information	255 (77.3)	175 (87.5)	430 (81.13)
Time-consuming	97 (29.4)	60 (30.0)	157 (29.62)
maintain relationship	150 (45.5)	118 (64.0)	268 (50.57)

a. Open-ended questions measured this item, and students were able to choose more than one or two answers to this question

### Psychometric properties of the scale

#### Face validity

The quantitative face validity indicated that the impact score was > 1.5 for the whole item. Regarding the qualitative face validity, participants represented slight alterations in the phrasing of some items for better elucidation.

#### Content validity

Assessing the tool qualitatively indicated that the whole criteria, including scaling of the questionnaire, grammar, allocating items, and phrasing, were appropriately chosen. The CVR and CVI values of the whole 33 items of the C-19MLs were respectively 0.77 and 0.94.

#### Construct validity phase

EFA.

**Fig. 2:**
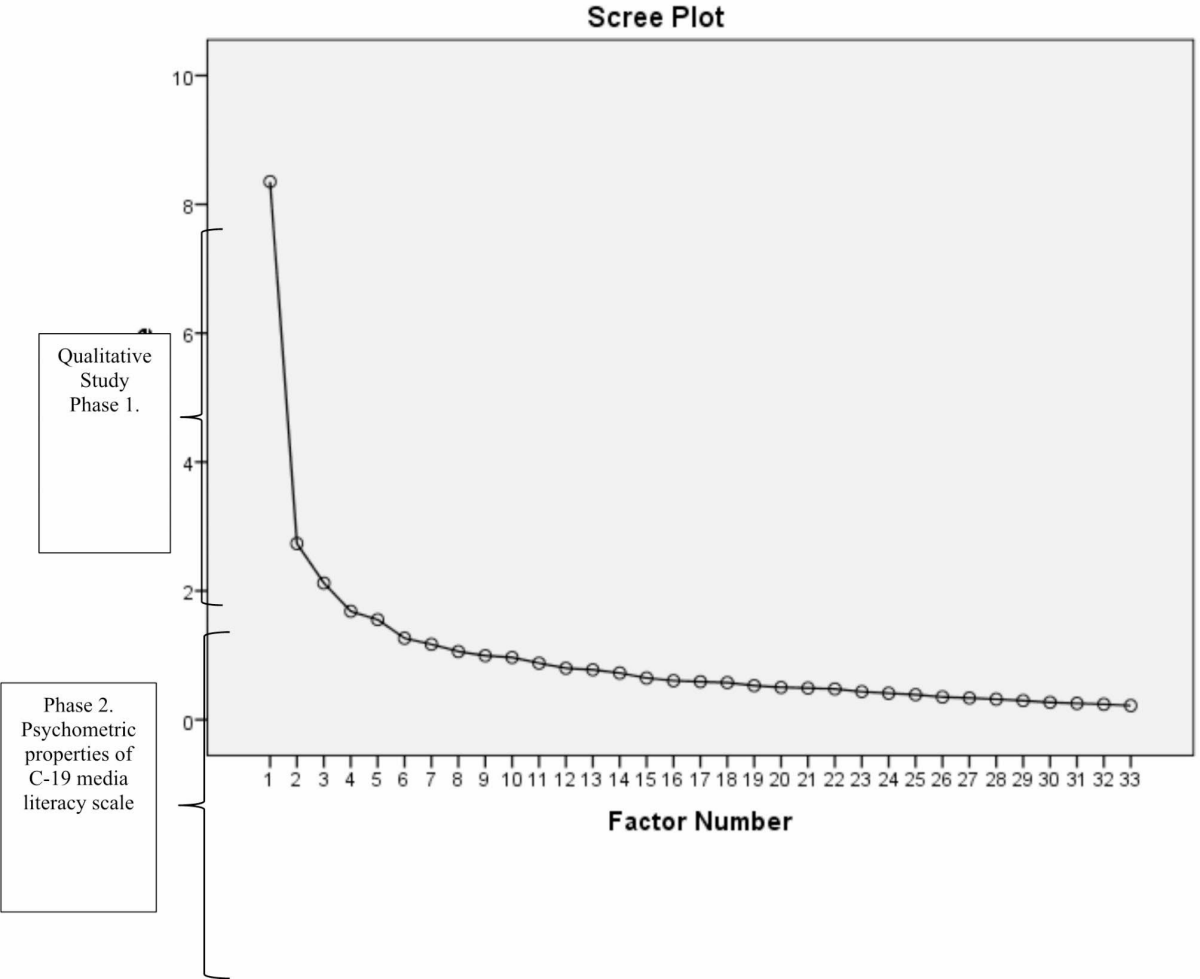
Primary EFA Scree plot of C-19MLs

Primary EFA results as presented in the following as Bartlett’s and KMO test indicated the appropriateness of the data for factor analysis (χ2 of 3978.533, KMO index of 0.86, df of 528, *P* < .001), approving the suitability of the factor model. These two tests revealed the appropriateness of the respondents’ data for EFA, which was conducted on the 33 items of the C-19ML scale by the highest likelihood process with Promax rotation. Based on primary exploratory factor loadings of items and the scree scheme (Fig. 2), eight factors were extracted, reporting an eigenvalue of higher than 1, accounting for 59.273% of the variance.

After careful assessment, some items were omitted for the following reasons:

- There was an item with low commonalities (e.g., less than 0.2) that were not highly correlated with one or more factors.
- Item loading on five items did not satisfy the expected threshold.
- There were three - factors with two or one item which, based on conceptual interpretability, failed to contribute meaningfully to any of the other factors or were ineffective to the items conjugated to other factors conceptually. Thus, in this process, it was decided to omit, and the other hand optimizes scale length.

Thus, item 24 had communalities = 0.16 < 0.2; five items [11, 15, 17, 22, and 23] were eliminated from the model because the factor loadings were less than 0.4. Furthermore, three factors [5–8] were deleted with fewer than three items ( 1, 2, 3, 4, 32, 33).

**Fig. 3:**
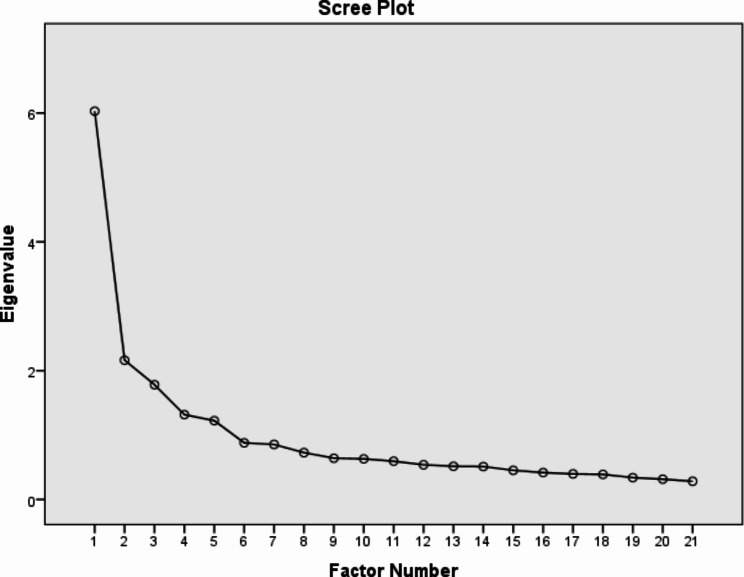
Final EFA Scree plot of C-19MLs

A final EFA was conducted to ensure that the factor solution does not change after deleting items and testing the factor structure [51]. The base of the result (χ2 of 2218.389, KMO index of 0.85, df of 210, *P* < .001), five factors were extracted, reporting eigenvalue of higher than one accounting for 58.41% of the variance with 21 items, remained Table 2, (Fig. 3).

**Table 2:** The final exploratory Results of Covid-19MLs with **five factors**

Item		F1		F2		F3	F4	F5
The WHO (World Health Organization) is among the constructedness of credible messages about Covid-19.		**0.730**		-0.016		0.080	-0.035	0.039
Organization and administration of health community services, the Ministry of Health, and medical universities are among the constructedness of credible messages about COVID-19.		**0.831**		− 0.002		0.009	0.066	− 0.088
Experienced specialists in infectious diseases and active health associations are among the constructedness of credible messages about COVID-19.		**0.679**		0.044		− 0.044	0.019	0.046
Sanitary ware producers and industrial and domestic disinfectant makers are among the constructedness of fake media coronavirus Messages.		0.029		**0.509**		0.010	− 0.102	0.043
Profiteering advertising companies are among the Constructedness of fake media coronavirus Messages.		0.054		**0.674**		− 0.027	0.004	0.058
Beneficiary politicians are among the Constructedness of fake media coronavirus Messages.		− 0.049		**0.677**		0.045	0.099	− 0.071
Curious people are among the audience of fake media coronavirus Messages.		0.070		0.043		**0.707**	− 0.042	− 0.043
The audiences of fake media coronavirus Messages are individuals with obsessive-compulsive disorder personalities.		− 0.008		0.000		**0.758**	0.022	0.004
Unproductive people are among the audience of fake media coronavirus Messages.		− 0.017		− 0.016		**0.538**	0.009	0.074
Individuals with any level of awareness, information, and income are the audience of COVID-19 media messages.		0.177		0.053		− 0.104	**0.456**	0.054
Highlighted the consequences of the coronavirus disease, such as the daily number of deaths, illness, and improvement across the country, are used to attract the audience’s attention in COVID-19 media messages.		− 0.092		0.108		0.042	**0.634**	− 0.112
To attract the audience’s attention to COVID-19 media messages, frequently repeated in a variety of media and social media is used.		− 0.004		0.140		− 0.031	**0.613**	0.001
To attract the audience’s attention to COVID-19 media messages, represented in the form of video clips, animations, and visual charts.		0.011		− 0.001		0.044	**0.653**	0.023
Credible messages about COVID-19 often Teach simple preventive instructions for public health “Such as using frequent hands washed with ordinary soap and water and wearing a mask.		0.061		− 0.230		0.058	**0.732**	0.000
To attract the audience’s attention with credible messages about COVID-19, often use available, popular, and easy-to-use social network media such as Instagram or Telegram, WhatsApp or TV and Radio		− 0.002		− 0.028		− 0.072	**0.700**	0.060
In fake media coronavirus, messages often represent beliefs such as COVID-19 vaccines developed have become less effective.		− 0.115		0.041		0.120	0.208	**0.498**
In fake media coronavirus, messages often represent beliefs such as alcohol consumption to prevent the disease.		− 0.014		− 0.044		0.113	− 0.005	**0.525**
In fake media coronavirus, messages often represent beliefs such as claiming traditional and herbs ingredients to be useful for disease prevention, such as drinking ginger and cinnamon tea		− 0.015		− 0.026		− 0.094	0.093	**0.622**
In fake media coronavirus, messages often represent beliefs such as weakening the virus and achieving herd immunity.		0.013		0.011		− 0.127	− 0.064	**0.771**
In fake media coronavirus, messages often represent beliefs such as the presence of the virus in the fresh air and transmission by foods or bites.		− 0.021		0.014		0.092	− 0.005	**0.617**
In fake media, coronavirus messages often represent beliefs such as the effectiveness of Anti-viral and anti-inflammatory drugs for disease prevention.		0.092		0.039		0.026	− 0.108	**0.704**
Eigenvalue		5.777		2.088		1.781	1.361	1.259
Explained variance (%)		25.510		9.945		8.479	6.479	5.997
Cumulative variance (%)		27.510		37.456		45.935	52.414	58.410
Extraction Method: Maximum Likelihood. Rotation Method: Promax with Kaiser Normalization.								

The bold factor loading of items is related to its factor

F1: Constructedness of credible COVID-19 media messages, F2: Constructedness of fake media coronavirus Messages, F3: Fake media Coronavirus Messages audience, F4: Format and F5: lifestyles are represented in fake media coronavirus Messages; the bold factor loading of items is related to its factors

CFA.

**Fig. 4:**
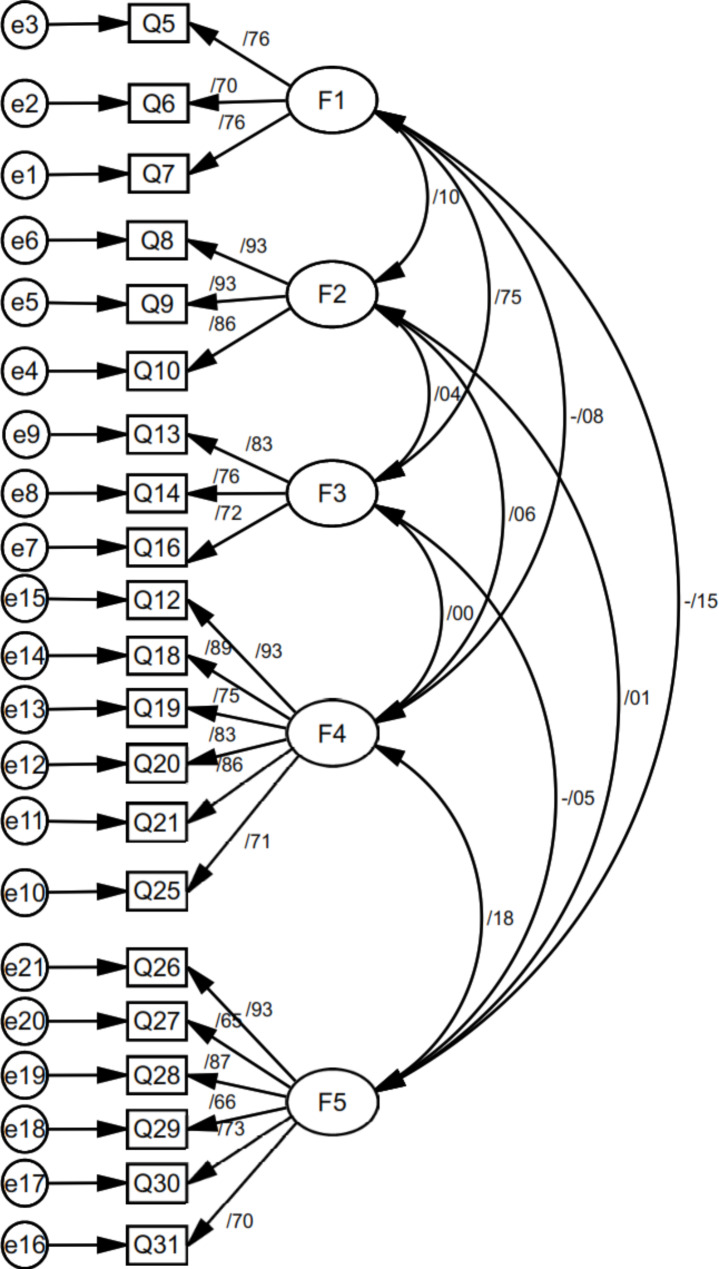
CFA of the C-19MLS questionnaire with Five-domain structure (F1: **Constructedness of credible Covid-19 media messages**, F2: **Constructedness of fake media coronavirus Messages**, F3: **Fake media Coronavirus Messages audience**, F4: **Format** and F5: **lifestyles are represented in fake media coronavirus Messages**)

The next step deals with confirming and validating the EFA-obtained factor structure utilizing the CFA (confirmatory factor analysis). According to the GOF (goodness-of-fit) indices, the studied model fits the standard accepted database appropriately. Thus, the CFA proves the model’s adequacy and the decent fitting of its structural model for the participants (Fig. 4). Table 3 represents the model fit indices.

**Table 3:** Measurement model-fit index

Measure	Recommended value	Result Value	Remark
Chi-square/degree of freedom	< 3	2.706	Good fit
Tucker Lewis Index	> 0.9	0.874	Good fit
Comparative Fit Index	> 0.9	0.893	Good fit
Goodness of Fit Index (GFI)	> 0.9	0.816	Good fit
Root mean square error of approximation	< 0.1	0.093	Good fit

#### Validity and reliability

Results of the proposed model fulfils the convergent validity.

To check the discriminant validity, the MSV was compared with AVE, and the square root of each dimension’s AVE was compared with the correlations for each pair of dimensions addressed by AVE and MSV (AVE > MSV) as presented in the correlation matrix Table 4, the MSV of all factors was lower than AVE, except factor one, which might be because of the low number of items (3 items of factor 1). So, for assurance about the reliability of the measurement instrument in addition to Cronbach’s alpha (CA), the reliability analysis was carried out in SPSS 24.0, and the results were presented in Table 5. It can be seen that the value of CA is greater than 0.60 for all the constructs. It can be concluded from the first step that the model is fit for carrying out SEM and path analysis as it meets all the validity issues. Also, internal consistency was used to evaluate the reliability. The mean ICC was 0.893 with a 95% confidence interval from 0.831 to 0.941 (F (600, 30) = 9.388, *P* < .001) (Table 5).

**Table 4:** Convergent validity and reliability

Factors	AVE	CR	MCV	MaxR(H)
Factor1^b^	0.555	0.789	0.557	0.791
Factor2^c^	0.826	0.934	0.009	0.941
Factor3^d^	0.595	0.815	0.557	0.825
Factor4^e^	0.690	0.930	0.032	0.946
Factor5^f^	0.583	0.892	0.032	0.927

Factor1^b^: **Constructedness of credible Covid-19 media messages**

Factor2^c^: **Constructedness of fake media coronavirus Messages**

Factor3^d^: **Fake media Coronavirus Messages audience**

Factor4^e^: **Format**

Factor5^f^: **lifestyles are represented in fake media coronavirus Messages**

**Table 5:** Cronbach’s alpha and ICC of the Factors of the **C-19MLS**

Factors	No of items	Mean	SD	Variance	Cronbach’s alpha	ICC^a^
Factor1^b^	3	11.23	2.459	6.047	0.802	0.878
Factor2^c^	3	10.42	2.363	5.585	0.655	0.736
Factor3^d^	3	9.97	2.415	5.832	0.713	0.681
Factor4^e^	6	24.16	3.397	11.540	0.807	0.857
Factor5^f^	6	23.77	3.930	15.447	0.803	0.679
TOTAL	21				0.863	0.893

ICC^a^: Intraclass Correlation Coefficient

Factor1^b^: **Constructedness of credible Covid-19 media messages**

Factor2^c^: **Constructedness of fake media coronavirus Messages**

Factor3^d^: **Fake media Coronavirus Messages audience**

Factor4^e^: **Format**

Factor5^f^: **lifestyles are represented in fake media coronavirus Messages**

However, the square root of each dimension’s AVE was bigger than the correlations for each dimension, indicating that the proposed factor structure possessed discriminate validity [4] (Table 6).

**Table 6:** Factor correlation and the squared root of AVE (on a diagonal)

Factors	Factor1^b^	Factor2^c^	Factor3^d^	Factor4^e^	Factor5^f^
Factor1^b^	**0.745**				
Factor2^c^	0.096	**0.909**			
Factor3^d^	0.740	0.045	**0.771**		
Factor4^e^	0.076	0.061	-0.004	**0.831**	
Factor5^f^	-0.155	0.013	-0.047	0.178	**0.746**

Factor1^b^: **Constructedness of credible Covid-19 media messages**

Factor2^c^: **Constructedness of fake media coronavirus Messages**

Factor3^d^: **Fake media Coronavirus Messages audience**

Factor4^e^: **Format**

Factor5^f^: **lifestyles are represented in fake media coronavirus Messages**

#### External validity

The independent sample t-test examined the external validity among different socio-demographic groups (Table 5). Based on the results, students were assured that lifestyles are represented in fake media coronavirus messages, format, constructedness of credible Covid-19 media messages, constructedness of fake media coronavirus messages, and the audience, respectively. Also, marital status and gender were considered for examining differences among dimensions. The result of the independent sample t-test indicated the constructedness of credible Covid-19 media messages dimension of the married student (M = 12.38, SD = 2.13) were higher than those of single students (M = 10.83, SD = 2.48), but it was not significant. The constructedness of a married student’s fake COVID-19 media messages dimension (M = 10.75, SD = 3.20) was higher than those of single students (M = 10.30, SD = 2.08), but it was not significant. The fake media coronavirus messages audience dimension of the married student (M = 10.50, SD = 2.51) were higher than those of single students (M = 9.78, SD = 2.41), but it was not significant. The lifestyles represented in fake media coronavirus messages dimension of the married student (M = 24.50, SD = 3.85) were higher than those of single students (M = 23.52, SD = 4.01), but it was insignificant.

The result of the independent sample t-test indicated the difference between the men and women was not significant, but the constructedness of credible COVID-19 media messages dimension of the female student (M = 11.42, SD = 2.30) was higher than that of male students (M = 10.20, SD = 3.27) but it was not significant. The constructedness of the female student’s fake Covid-19 media messages dimension (M = 10.46, SD = 3.20) was higher than those of single students (M = 10.30, SD = 2.08) but was not significant. The fake media coronavirus messages audience dimension of male students (M = 10.60, SD = 2.88) was higher than that of female students (M = 9.85, SD = 2.36), but it was insignificant. The lifestyles represented in fake media coronavirus messages dimension of male students (M = 24.00, SD = 6.04) were higher than those of female students (M = 23.73, SD = 3.56), but it was insignificant.

## Discussion

### Summary of findings

The study aimed to assess the validity and reliability of 21-item C-19ML. As indicated by the EFA, the C-19MLs is a 5-domain structure with five factors F1 (**Constructedness** of credible Covid-19 media messages) F2 (**Contractedness** of fake media coronavirus messages), F3 (Fake media coronavirus messages **Audience**), F4 (**Format**), and F5 (**Represented lifestyles** in fake media coronavirus Messages) [52].

**Constructedness of credible media coronavirus messages** dimension means Who create credible Covid-19 media messages?”. **Contractedness of fake media coronavirus messages** dimension means “Who create credible Covid-19 media messages?”. **Fake media coronavirus messages audience** dimension means “Who may deal with fake Covid-19 media messages”. **Format** dimension means “What creative techniques are used to attract my attention?“. meaning of **represented lifestyles** dimension is media have embedded value and point of view; “What lifestyle, value and point of view are presented in or omit from this message?” (“Supplementary file”).

### Validity

The validity is a fundamental feature of questionnaires aimed at detecting the ability of an instrument to measure the object through its design. Constructed validation is vital for determining a questionnaire’s validity, mainly in psychometrical subjects. The ideal process in this regard is factor analysis [53]. The main frameworks of the research questionnaires included 33 items. In this phase, the operation of EFA caused the elimination of 12 items from the primary questionnaire. Ultimately, a form with 21 items was categorized into a 5-domain structure.

Based on the final EFA with Promax rotation findings, extracting the 5-domain structure is possible along with explicit contractedness of credible Covid-19 media messages, contractedness of fake media coronavirus messages, fake media coronavirus messages audience, format, and lifestyles are represented in fake media coronavirus messages. The former research supported the 5-domain structures. Our findings in this part correspond to developing guidance. CML MediaLit Kit™ develops the educational philosophy of empowerment via education through some records and Internet sources articulating the concept, execution, and application of Media Literacy within the US educational system. Elizabeth Thoman (1994), the CML creator, made her fundamental paper “Skills and Strategies for Media Education” with this kit [21, 54, 55].

C-19MLs measure media literacy related to COVID-19 media messages. According to the qualitative analysis of exploring the experience of people’s COVID-19 Media Literacy, the last 21 items recognized that the scale was accomplished by the C-19MLs measurement [56].

Analyzing the KMO index and Bartlett’s Test of Sphericity indicated the adequacy of the sample size and satisfaction of the factor analysis [48, 57, 58]. Rejecting the null hypothesis of data Sphericity and confirming the KMO statistic were obtained in our study. The five factors accepted here could clarify 60.0% of the variance, and the most pronounced variations were associated with the supposed power. Correspondingly, Koc et al. assessed the New Media Literacy Scale (NMLS) and reported that varimax orthogonal rotation presented the ultimate four-factor model, with the remaining 35 items accounting for 55% of the total variance [30]. We made our C-19MLs based on Primack et al.’s scale for the theoretically similar smoking media literacy [22]; based on their theoretical method of smoking media literacy, we believe the same framework might apply to COVID-19. On the other hand, both Bier et al. and Ashley et al. found a positive association between general media literacy and smoking media literacy regarding the same underlying theoretical framework [25, 59].

Content validity is strong, by which scale items were oriented by a precisely designed framework integrating media literacy models with the greatest acceptability. Furthermore, this strength is associated with items in the resultant scale representing the framework’s core concepts. These findings provided evidence that both factors are statistically applicable and equivalent to measuring media literacy (ML). The only difference between factors in C-19MLs and other scales of Media Literacy. In explaining, it can be said that a special issue of the C-19MLs presents an alternative account of the relationship between factors in media literacy and health education and health promotion themes. That is, C-19MLs is a specific measuring scale in media literacy, and until now, there aren’t specific media literacy scales for measuring individuals.

The present study’s finding examined the external validity, in which all dimensions were different among marital status and gender., mean scores of all dimensions in married students were higher than in singles. Chang et al.‘s study noted an increase in parents’ search for medical information and an increase in e-health literacy, which could be consistent with the present study’s findings [60]. Also, previous evidence found gender differences in computer and information literacy [61]. Notably, studies recommend that boys and girls may perform differently on computer and information literacy skills, and these skills require learning in higher competencies, especially in emergencies such as the COVID-19 pandemic [62, 63].

### Reliability

The reliability represents the stableness and consistency of an instrument’s constructs, indicating the questionnaire’s measuring accuracy [36]. The last 21-item scale possesses an excellent internal consistency, with a Cronbach’s alpha of 0.86, indicating the acceptable reliability of the suggested questionnaires. Primack et al. performed a study with a Cronbach’s alpha of 0.87; the last scale with 18 items had exceptional internal consistency [22]. Ashrafi-rizi et al. (2014), investigating the Media Literacy Scale, reported satisfactory reliability with a Cronbach’s alpha of 0.89 [64].

Though the ICC level was desired in all domain structures, that means satisfactory quantities of ICC; nevertheless, an excellent internal consistency existed between these domain structures with a Cronbach’s alpha of 0.89. Thus, it is suggested to consider this fact in approaching studies.

### The strengths and limitations

Regardless of some strength in our investigation, such as compared with previous tools, the newly developed C-19MLs is a multidimensional specific media literacy scale related to emerging diseases.

Also, this scale focuses on specific media literacy in developing countries among college students. On the other hand, other scales of ML emphasize that media literacy is both an understanding producer and a consumer of media content, or media literacy is generally managing consuming media, which these notions are traditional.

Also, with the appearance of new media technology along with emerging diseases, critical thinking about new dimensions such as creators of media massages, methods of persuading the audience by creators of media massages [65], and presenting/omitting new patterns in lifestyle by misinformation and fake news [66] could be useful; because these typologies of information can play an essential role in teaching proper health information-seeking behavior when facing with the crises which in other scales to be neglected.

Some limitations exist likewise; first, this work was conducted by a student of Hamadan University of Medical Sciences, which suggested future studies could be conducted in other areas or countries to test the external validity.

Second, it’s impractical to ensure the utilization of the conclusions to the populations in numerous geographical areas or another context because this scale measures COVID-19 media literacy in developing countries. So, it could be validated in future studies in developed countries. Thirdly, we deleted the items based on EFA results; therefore, we suggested adding additional items in future studies.

Also, future studies must compare studies about C-19MLs in students after and before COVID-19 and during seasonal influenza [67].

## Conclusions

Concisely, C-19MLs is a scale with reliability and validity for evaluating COVID-19 media literacy among students. Validation assessments with various longitudinal designs and populations should be vitally aimed at purifying, adjusting, or confirming the C-19MLs as an additional, complementary media literacy (ML) tool. Moreover, suggestions for future work in defining and assessing the field of health investigation and media literacy. This indicates the significance for educators and stakeholders to realize the vital participating individuals in the new media ecology and new ‘Infomedia’ ecosystems for empowering people, especially in online health information searches in the youth. Also, this scale could be applied for designing interventional strategies, particularly in cyberchondria, “digital syndrome” prevention in societies.

### Electronic supplementary material

Below is the link to the electronic supplementary material.

Supplementary Material 1

## Data Availability

The datasets used and analyzed during the current study are available from the corresponding author upon reasonable request.

## Declarations

Competing of Interest:

## Abbreviations

ML: Media literacy
C-19MLs: The COVID-19 Media Literacy Scale
ICC: Intraclass Correlation Coefficient
CFA: Confirmatory Factor Analyses
EFA: Exploratory Factor Analyses
CVR: Content Validity Ratio
CVI: Content Validity Index
