# Supplementary material for: Developing and validation of COVID-19 media literacy scale among students during the COVID-19 pandemic
Source: BMC Psychol. 2023 Oct 6;11:315. doi: 10.1186/s40359-023-01353-6 (PMC10559652; doi:10.1186/s40359-023-01353-6)
Supplement: Supplementary file 1 — Supplementary Material 1 [file 40359_2023_1353_MOESM1_ESM.docx]

**Appendix**

**Covid-19 Media Literacy Scale (C-19MLS) in Students**

| **How much do you agree with the following statements?** | **completely disagree**  **1** | **Disagree**  **2** | **no idea**  **3** | **Agree**  **4** | **completely agree**  **5** |
| --- | --- | --- | --- | --- | --- |
| Constructedness of credible Covid-19 media messages |  |  |  |  |  |
| 1. **The WHO, (World Health Organization) is among the constructedness of credible messages about Covid-19.** |  |  |  |  |  |
| 1. **Organization and administration of health community services, ministry of health and medical universities are among the constructedness of credible messages about Covid-19.** |  |  |  |  |  |
| 1. **Experienced specialists in the field of infectious diseases and active health associations are among the constructedness of credible messages about Covid-19.** |  |  |  |  |  |
| Contractedness of fake media coronavirus Messages |  |  |  |  |  |
| 1. **Sanitary ware producers, industrial and domestic disinfectants makers** **are among the constructedness of fake media coronavirus Messages.** |  |  |  |  |  |
| 1. **Profiteering advertising companies are among the Constructedness of fake media coronavirus Messages.** |  |  |  |  |  |
| 1. **Beneficiary politicians are among the Constructedness of fake media coronavirus Messages.** |  |  |  |  |  |
| Fake media Coronavirus Messages audience |  |  |  |  |  |
| 1. **Curious people are among the audience of fake media coronavirus Messages.** |  |  |  |  |  |
| 1. **The audiences of fake media coronavirus Messages are individuals with obsessive-compulsive disorder personality.** |  |  |  |  |  |
| 1. **Unproductive people are among the audience of fake media coronavirus Messages.** |  |  |  |  |  |
| Format |  |  |  |  |  |
| 1. **The individuals with any level of awareness, information and income are the audience of Covid-19 media messeges.** |  |  |  |  |  |
| 1. **Highlighted the consequences of the coronavirus disease such as daily number of deaths, illness and improvement across country is used to attract the audience’s attention in Covid-19 media messeges.** |  |  |  |  |  |
| 1. **To attract the audience’s attention of Covid-19 media messeges, frequently repeated in a variety of media and social media is used.** |  |  |  |  |  |
| 1. **To attract the audience’s attention of Covid-19 media messeges, represeanted in form of video clips, animations and visual charts.** |  |  |  |  |  |
| 1. **In credible messages about Covid-19 often Teach simple preventive instructions for public health "Such as using frequent hands washed with ordinary soap and water, wearing a mask.** |  |  |  |  |  |
| 1. **To attract the audience’s attention in credible messages about Covid-19 often use available, popular and easy-to-use social network media such as Instagram or Telegram, WhatsApp or TV and Radio** |  |  |  |  |  |
| Lifestyles are represented in fake media coronavirus Messages |  |  |  |  |  |
| 1. **In fake media coronavirus Messages often represent beliefs such as COVID-19 vaccines developed have become less effective.** |  |  |  |  |  |
| 1. **In fake media coronavirus Messages often represent beliefs such as alcohol consumption to prevent the disease.** |  |  |  |  |  |
| 1. **In fake media coronavirus Messages often represent beliefs such as claiming traditional and herbs ingredients to be useful for disease prevention such as drinking ginger and cinnamon tea** |  |  |  |  |  |
| 1. **In fake media coronavirus Messages often represent beliefs such as the weakening of the virus and achieve herd immunity.** |  |  |  |  |  |
| 1. **In fake media coronavirus Messages often represent beliefs such as the presence of the virus in the ambient and transmission food or bites.** |  |  |  |  |  |
| 1. **In fake media coronavirus Messages often represent beliefs such as** **the effectiveness of Anti-viral and anti-inflammatory drugs for disease prevention.** |  |  |  |  |  |
